# Identification of hMex-3A and its effect on human bladder cancer cell proliferation

**DOI:** 10.18632/oncotarget.18050

**Published:** 2017-05-22

**Authors:** Ying Huang, Chao Fang, Jing-Wen Shi, Yu Wen, Da Liu

**Affiliations:** ^1^ Department of Ultrasound, Shengjing Hospital of China Medical University, Shenyang 110004, China; ^2^ Department of Histoembryology, China Medical University, Shenyang 110000, China; ^3^ Department of Orthopedics, Shengjing Hospital of China Medical University, Shenyang 110004, China

**Keywords:** hMex-3A, RNA-binding protein, cell proliferation, bladder cancer

## Abstract

In this study, *hMex-3A* was selected from TCGA database as a research object to observe the effects of small interfering RNA (siRNA) targeting *hMex-3A* on the biological activities of human bladder cancer and explore its mechanism for the first time. In this study, there were 2 groups including negative control group and *hMex-3A*-siRNA-transfected cells group for 5637 and T24 cell lines, respectively. After bladder cancer cells were transfected with the interference RNA sequence, proliferation of transfected cells were assessed by Celigo Cell Counting, and apoptosis were detected by flow cytometry. The knockdown rate of *hMex-3A* was 74% in 5637 cells and 68% in T24 cells after RNA interference. In addition, Celigo Cell Counting indicated that cell viability was significantly lower in *hMex-3A*-siRNA-transfected cells group (2196/well) than in negative control group (6777/well) (*P* < 0.05), but T24 cells did not show statistical significance between *hMex-3A*-siRNA-transfected cells group (5799/well) and negative control group (7899/well) (*P* >0.05). Flow cytometer showed that apoptosis was the highest and cells were significantly blocked after cells were transfected in *hMex-3A*-siRNA-transfected cells group in 5 days later (*P* < 0.05). Mex-3A protein was detected in bladder carcinoma sections with a mean staining intensity of 7.06±2.60. Mex-3A protein expression was significantly higher in cancerous tissue than in para-cancerous tissue (*P* <0.05). Our study suggested that siRNA targeting *hMex-3A* could markedly inhibit cell proliferation and promote apoptosis in 5637 cells. These might have significant implications to bladder carcinogenesis and serve as a potential target for the treatment of bladder cancer.

## INTRODUCTION

It is now increasingly clear that post-transcriptional regulation is a fundamental mechanism guiding gene expression in higher eukaryotic cells, such as the core of normal cellular processes, and cancer initiation and development. As we know, RNA maturation, localization, translation and stability are dependent on the cooperation between cis-regulatory elements and trans-acting factors, such as non-coding RNAs and RNA-binding proteins (RBPs) [1–3].

RNA-binding proteins of the evolutionarily conserved Mex-3 family are mediators of post-transcriptional regulation in different organisms, and involved in diverse physiological settings. Mex-3 proteins are associated with diseases, particularly cancer. In humans, Mex-3 was identified and was found to have four homologous genes, Mex-3 A-D [4, 5]. It is important to ascertain their contribution to carcinogenesis and evaluate their potential as markers of cancer progression or prognosis [4].

Bladder cancer is one of the most common types of urinary system malignant tumors, with an estimated 429,800 new cases with bladder cancer and 165,100 deaths due to bladder cancer in 2012 worldwide. In the present study, one of the four proteins of *hMex-3*, *hMex-3A*, was selected to investigate its function in bladder cancer development and progression for the first time and explored its role in bladder cancer development.

## RESULTS

### Analysis of TCGA database and Mex-3A gene screening

The information about RNAseq was downloaded from TCGA database according to bladder cancer-related key words. Using barcode, the original data of 19 paired-samples, in which 2 paired-samples had no pathological information, were found (Table 1). In TCGA database, the difference in Mex-3A expression between cancerous tissue and para-cancerous tissue was reflected by FC (ratio of expression level in cancerous tissue to expression level in para-cancerous tissue) and *P*-value (statistical analysis model which is used to determine whether a statistic is consistent with null hypothesis) (Table 2).

**Table 1:** The original data of 19 paired-samples of bladder carcinoma from TCGA database

No.	Sample name	Method initial path dx	Tissue source site	Tumor tissue site	Histological type	Neoplasm histologic grade
1	TCGA-BL-A13J	Transurethral resection (TURBT)	BL	Bladder	Muscle invasive urothelial carcinoma (pT2 or above)	High grade
2	TCGA-BT-A20N	Transurethral resection (TURBT)	BT	Bladder	Muscle invasive urothelial carcinoma (pT2 or above)	High grade
3	TCGA-BT-A20Q	Transurethral resection (TURBT)	BT	Bladder	Muscle invasive urothelial carcinoma (pT2 or above)	High grade
4	TCGA-BT-A20R	Other method, specify:	BT	Bladder	Muscle invasive urothelial carcinoma (pT2 or above)	High grade
5	TCGA-BT-A20U	Other method, specify:	BT	Bladder	Muscle invasive urothelial carcinoma (pT2 or above)	High grade
6	TCGA-BT-A20W	Other method, specify:	BT	Bladder	Muscle invasive urothelial carcinoma (pT2 or above)	High grade
7	TCGA-BT-A2LA	Other method, specify:	BT	Bladder	Muscle invasive urothelial carcinoma (pT2 or above)	High grade
8	TCGA-BT-A2LB	Transurethral resection (TURBT)	BT	Bladder	Muscle invasive urothelial carcinoma (pT2 or above)	High grade
9	TCGA-CU-A0YN	Transurethral resection (TURBT)	CU	Bladder	Muscle invasive urothelial carcinoma (pT2 or above)	High grade
10	TCGA-CU-A0YR	Transurethral resection (TURBT)	CU	Bladder	Muscle invasive urothelial carcinoma (pT2 or above)	High grade
11	TCGA-GC-A3BM	Transurethral resection (TURBT)	GC	Bladder	Muscle invasive urothelial carcinoma (pT2 or above)	High grade
12	TCGA-GC-A3WC	Endoscopic biopsy	GC	Bladder	Muscle invasive urothelial carcinoma (pT2 or above)	High grade
13	TCGA-GD-A2C5	Transurethral resection (TURBT)	GD	Bladder	Muscle invasive urothelial carcinoma (pT2 or above)	High grade
14	TCGA-GD-A3OP	Transurethral resection (TURBT)	GD	Bladder	Muscle invasive urothelial carcinoma (pT2 or above)	High grade
15	TCGA-GD-A3OQ	Transurethral resection (TURBT)	GD	Bladder	Muscle invasive urothelial carcinoma (pT2 or above)	High grade
16	TCGA-K4-A3WV	Other method, specify:	K4	Bladder	Muscle invasive urothelial carcinoma (pT2 or above)	High grade
17	TCGA-K4-A54R	Transurethral resection (TURBT)	K4	Bladder	Muscle invasive urothelial carcinoma (pT2 or above)	High grade
18	TCGA-GC-A6I3					
19	TCGA-K4-A5RI					

**Table 2:** Differential expression of Mex-3A in cancerous tissue and para-cancerous tissue from TCGA database

ID	Gene symbol	FC	P-value	Total sample	Sample unchanged	Sample up	Sample down
92312	MEX3A	10.737	3.47E-14	19	2	17	0

### Identification of hMex-3A in human bladder cancer cell lines 5637 and T24

**Figure 1:**
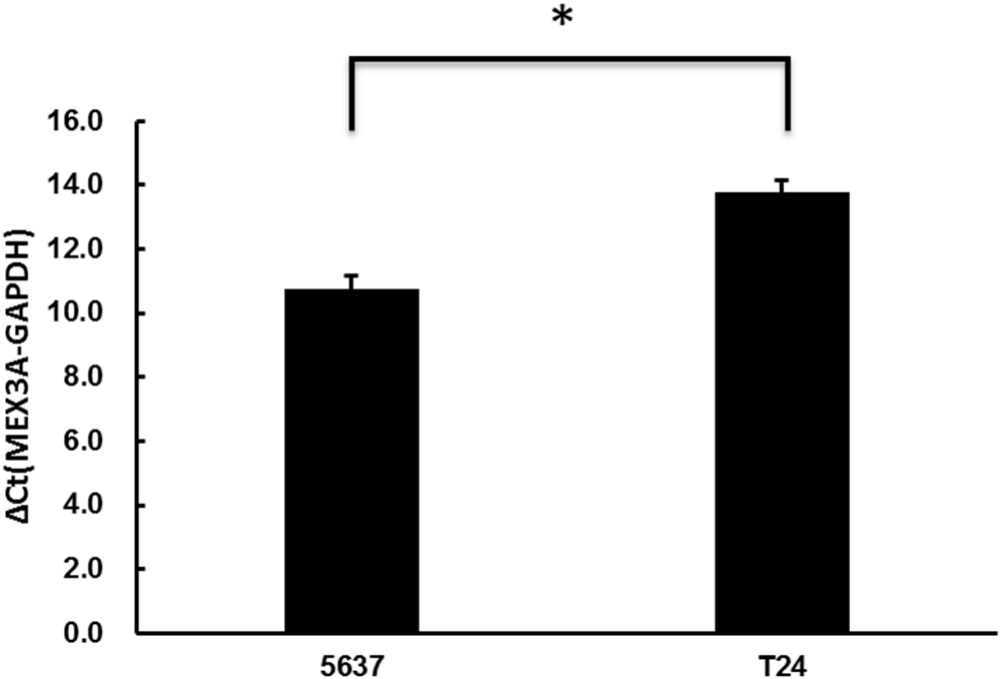
*hMex-3A* mRNA expression by real-time quantitative PCR in 5637 and T24 cells Notes: The mRNA level of each sample is normalized to that of *GAPDH* using the 2-ΔCt method prior to comparative analysis. ΔCt is equal to the difference between the ΔCts of *hMex-3A* and *GAPDH*. *hMex-3A* expression is significantly higher *in 5637* cells than in T24 cells (*P* <0.05). * indicates *P* <0.05.

We measured *hMex-3A* mRNA expression by real-time quantitative PCR in 5637 and T24 cells. The mRNA level of each sample was normalized to that of *GAPDH* prior to comparative analysis using the 2-ΔCt method. ΔCt is equal to the difference between the ΔCts of *hMex-3A* and *GAPDH*. The average ΔCts were 10.76±0.397 and 13.77±0.361 in 5637 and T24 cells, respectively (Figure 1). *hMex-3A* level in *5637* cells was significantly higher than that in T24 cells (*P* <0.05).

### Infection efficiency after RNA interference and transfection

**Figure 2:**
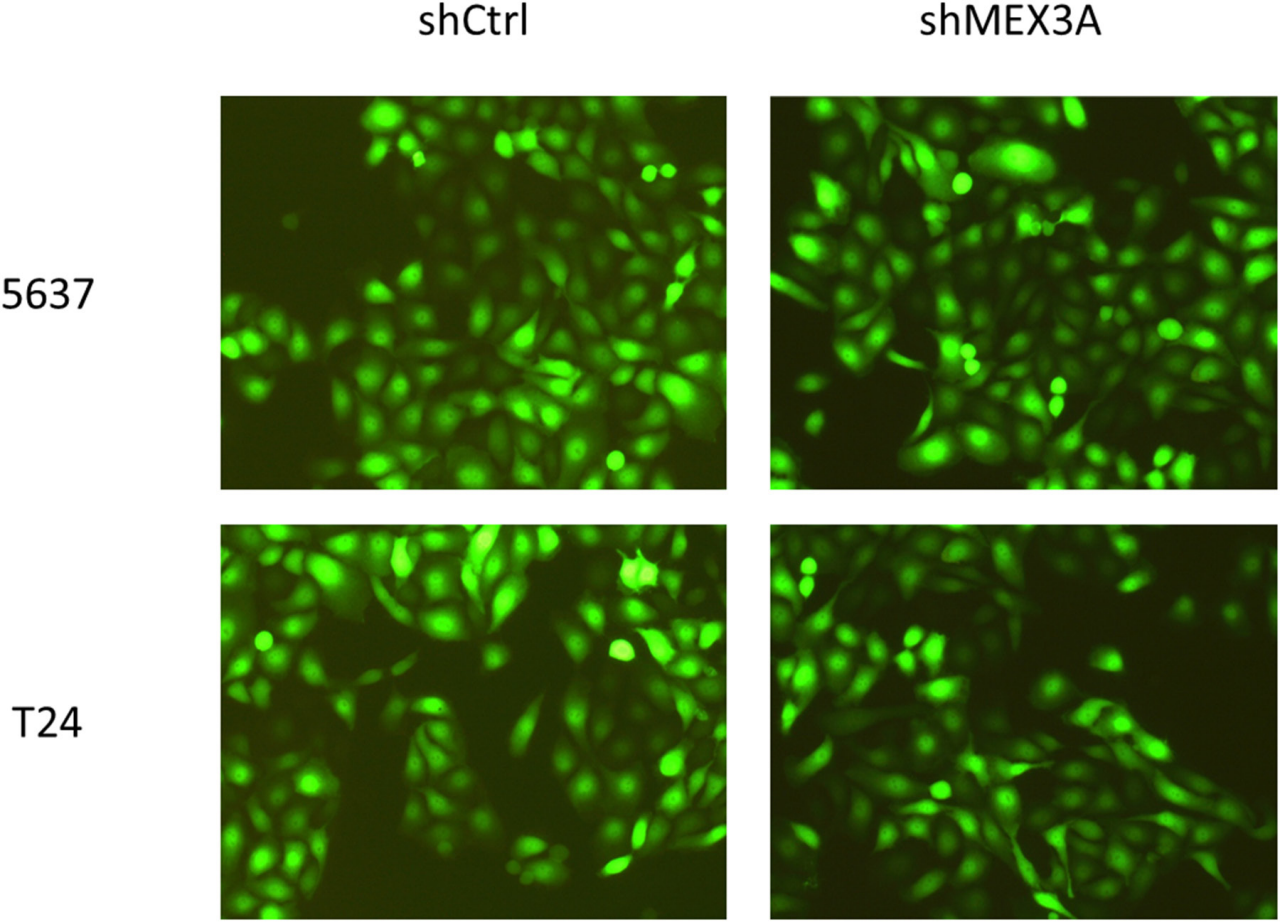
5637 and T24 cells infected by shCtrl and shMex3A slow virus for 72 hours under fluorescence microscope Notes: 5637 (upper panels) and T24 cells (lower panels) are infected by shCtrl (upper panels) and shMex3A slow virus (lower panels) for 72 hours respectively and the results show that the fluorescent protein expression is about 80% by fluorescence microscope (green staining), while the cells shape keep normal mainly ×100.

5637 and T24 cells were infected by shCtrl and shMex3A slow virus 72 hours respectively, and the results showed that the fluorescent protein expression (green staining) is about 80% by fluorescence microscope, while the cells shape kept normal mainly (Figure 2).

### Quantitative real-time PCR to determine Mex-3A mRNA levels in post-transfected cells

**Figure 3:**
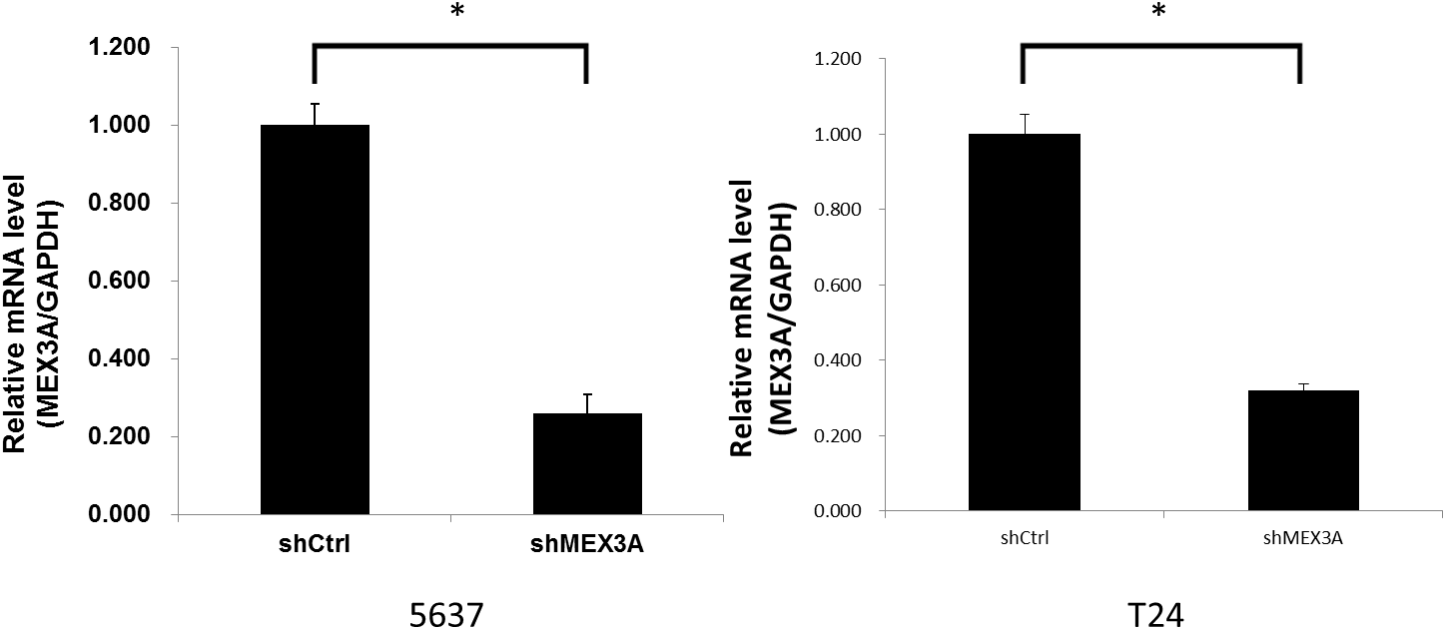
*hMex-3A* mRNA levels in *hMex*-3A-siRNA-transfected cells and non-specific siRNA-transfected cells by quantitative real-time PCR Notes: The mRNA level of each sample is normalized to that of *GAPDH* using the 2-ΔCt method prior to comparative analysis. The relative mRNA levels (*hMex-3A*/*GAPDH*) in shCtrl and shMex3A groups were 1.001±0.053 and 0.260±0.049 in 5637 cells, and were 1.001±0.047 and 0.319±0.038 in T24 cells, respectively (all *P* <0.05). The knockdown rate of *hMex-3A* was 74% and 68% in 5637 and T24 cells, respectively. * indicates *P* <0.05.

We measured *hMex-3A* mRNA expression by real-time quantitative PCR in 5637 and T24 cells after RNA interference. The mRNA level of each sample was normalized to that of *GAPDH* prior to comparative analysis using the 2-ΔCt method. The relative mRNA levels (*hMex-3A*/*GAPDH*) in shCtrl and shMex3A groups were 1.001±0.053 and 0.260±0.049 in 5637 cells, and were 1.001±0.047 and 0.319±0.038 in T24 cells, respectively (Figure 3) (all *P* <0.05). The knockdown rate of *hMex-3A* was 74% and 68% in 5637 and T24 cells, respectively.

### Silencing of hMex-3A by RNA interference inhibits bladder cancer cell growth

**Figure 4:**
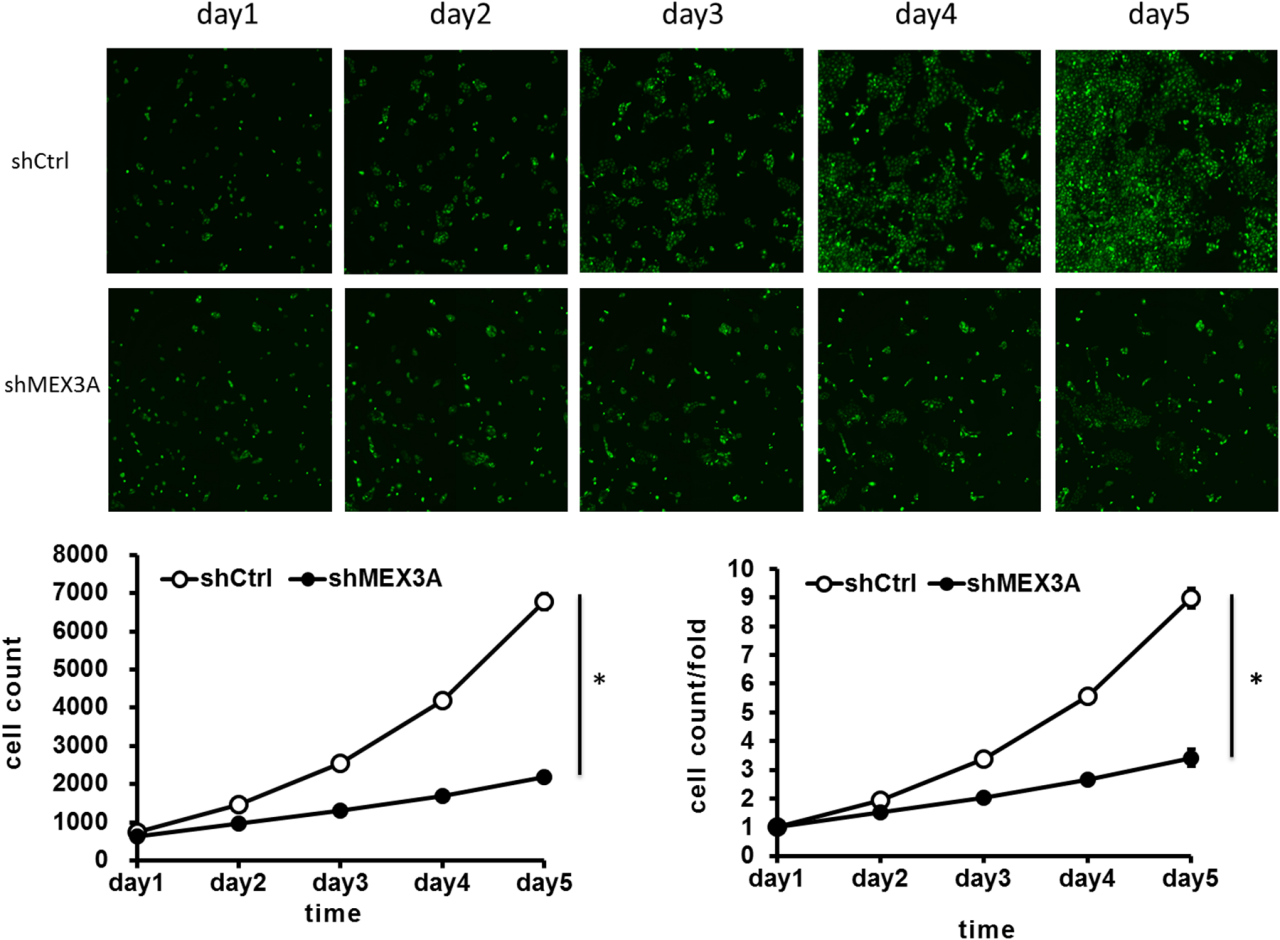
*hMex-3A* knockdown inhibits 5637 cell growth as compared with control group Notes: 5637 cells are infected by shCtrl and shMex3A slow virus for 5 days, and fluorescence microscope (green staining) shows that the cell count is increased in a time-dependent model, but the increase rates are different at different time points. Celigo Cell Counting indicated that cell growth is slower in shMex3A group (middle panels) than in shCtrl group(upper panels). In addition, the cell count and count fold curves show significant differences between shMex3A group and shCtrl group (*P* <0.05). * indicates *P* <0.05.

To assess whether *hMex-3A* has an effect on cell growth, RNA interference against *hMex-3A* was carried out in the bladder cancer cell lines 5637 and T24. 5637 cells were infected by shCtrl and shMex3A slow virus for 5 days, and fluorescence microscope showed that the cell count was increased in a time-dependent model, but the increase rates were different at different time points. Celigo Cell Counting indicated that 5637 cell growth was slower in *hMex-3A*-siRNA-transfected group (2196/well) than in negative control group (6777/well) (*P* < 0.05) in 5 days later. In addition, the cell count and count fold curves showed significant differences between of *hMex-3A*-siRNA-transfected cells group and negative control group in 5637 cells (Figure 4) (*P* <0.05).

**Figure 5:**
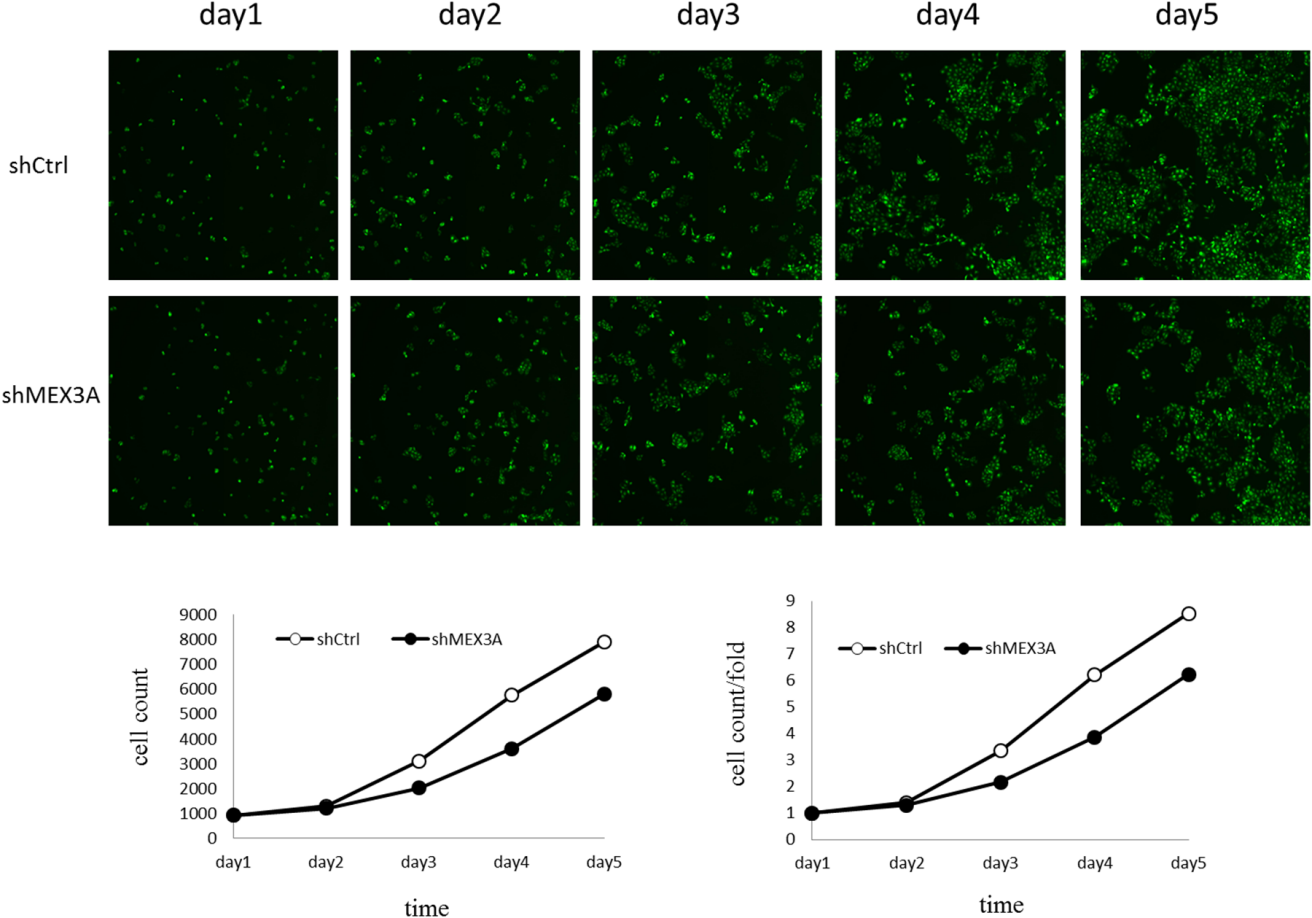
The effect of *hMex-3A* knockdown on T24 cell growth as compared with control group Notes: Cell count and count fold curves show no significant differences between shMex3A group and shCtrl group in T24 cells (*P* >0.05).

Cell growth was slower in *hMex-3A*-siRNA-transfected group (5799/well) than in negative control group (7899/well) in T24 cells 5 days later. However, there was no significant difference between the two groups (*P* > 0.05). The cell count and count fold curves showed no significant differences between of *hMex-3A*-siRNA-transfected group and negative control group in T24 cells (Figure 5) (*P* >0.05). It implied that the effect of *hMex-3A* on cell growth is dependent on cell type.

### Cell apoptotic analysis

**Figure 6:**
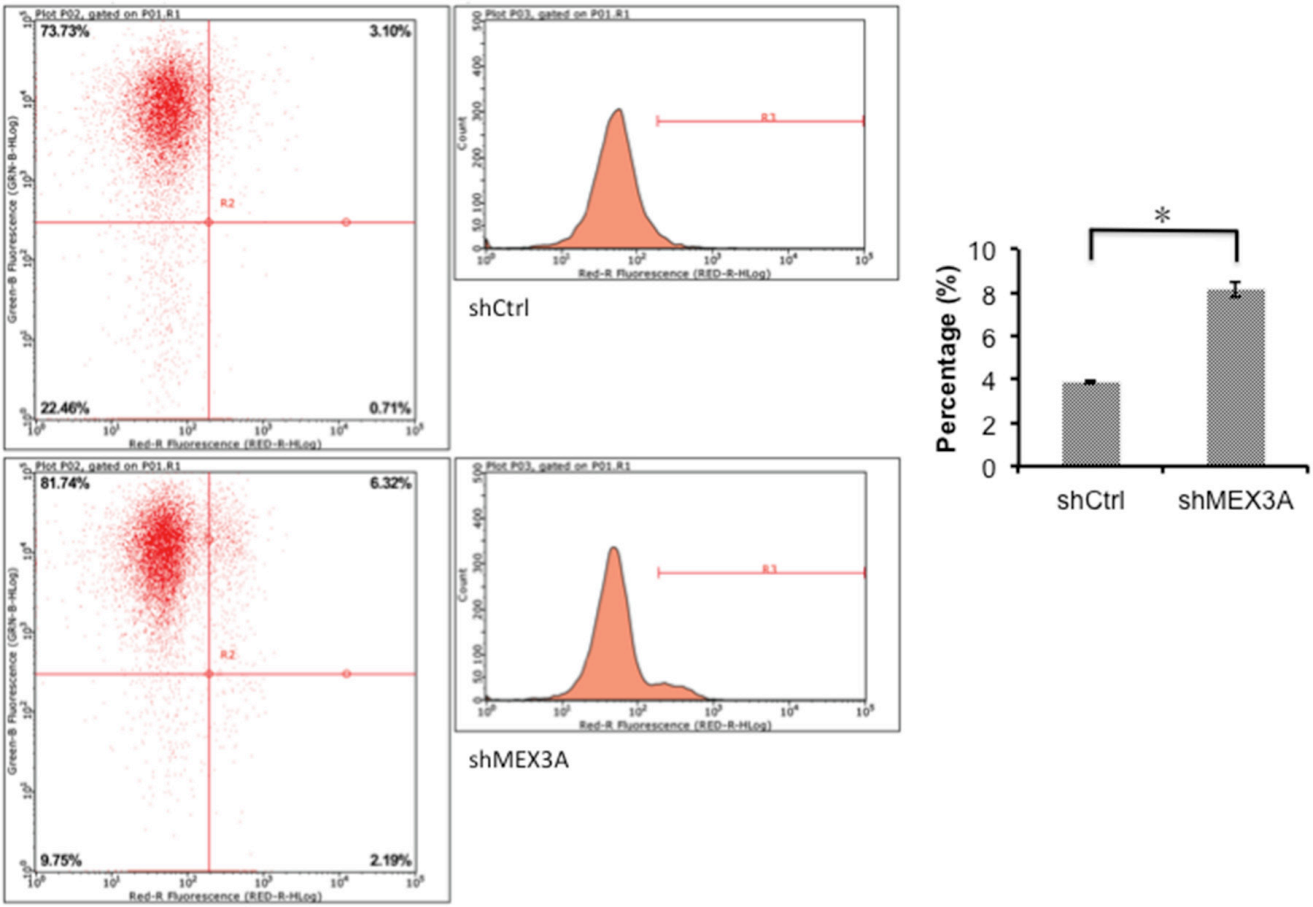
5637 cell apoptosis analyzed by flow cytometry Notes: Cells are transfected with si-psc and si-NC for 5 days, apoptosis is analyzed by flow cytometry. The resulting data indicate that the cell apoptosis is significantly higher in shMex3A group than in shCtrl group (*P* <0.05). * indicates *P* <0.05.

Since *hMex-3A* level in *5637* cells was significantly higher than that in T24 cells (*P* <0.05) and the effects of siRNA targeting *hMex-3A* on growth inhibition was not marked in T24 cells, we only chose 5637 cell line for the detection of apoptosis. To investigate the apoptosis in 5637, cells were analyzed by flow cytometry 5 days after transfection with si-psc. The apoptosis rates were 3.88±0.0651 and 8.14±0.3579 in shCtrl and shMex3A groups, respectively (Figure 6) (*P* <0.05).

### hMex-3A expression in human bladder cancer

**Figure 7:**
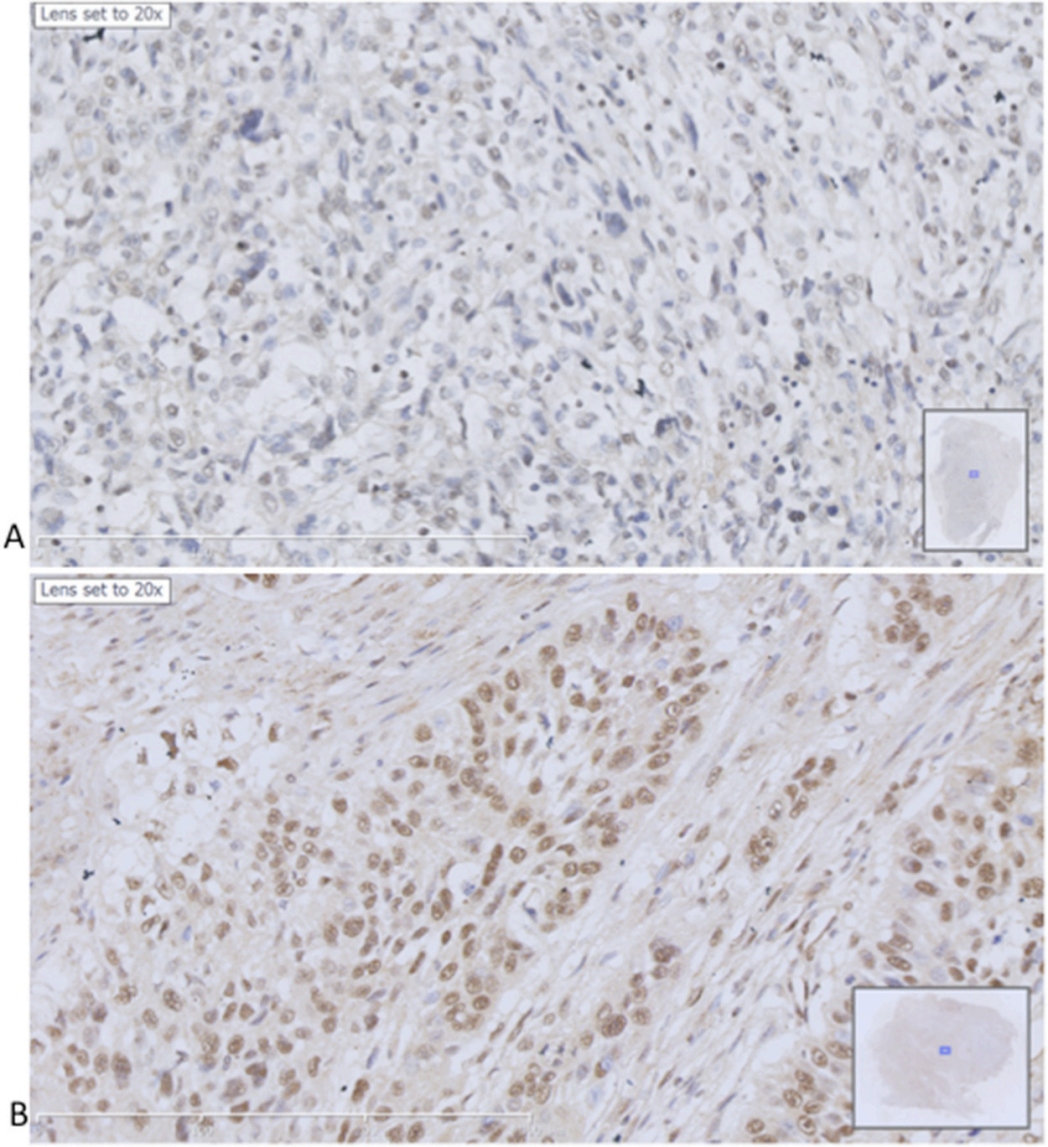
Immunohistochemical staining of hMex-3A in human bladder cancers **(A)** hMex-3A expression in para-cancerous tissue with sparse brown particles in cytoplasm and nuclei ×200. **(B)** hMex-3A expression in cancerous tissue with concentrated brown particles in cytoplasm and nuclei ×200.

**Figure 8:**
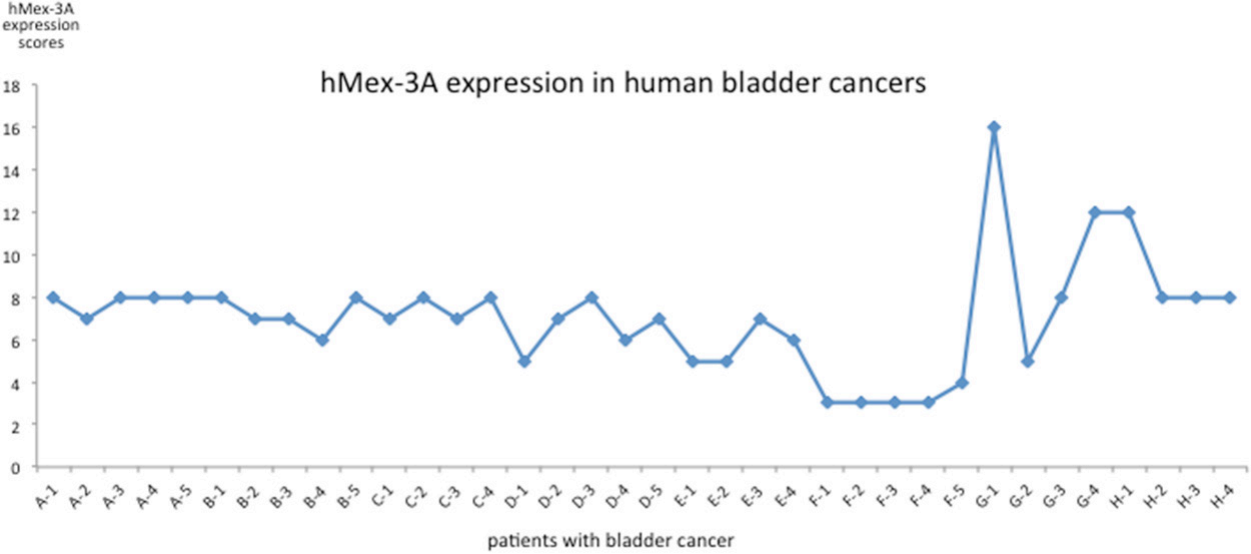
hMex-3A expression in human bladder carcinoma hMex-3A protein expression is detected in tissue sections from 8 patients with bladder carcinoma. The mean of total score including staining intensity and staining rate is 7.0556±2.5961. Low expression is found in 2 samples and high expression in 6 samples.

We collected 8 samples of bladder carcinoma including one sample from non-invasive urothelial carcinoma (high-level) and 7 samples from invasive urothelial carcinoma. Mex-3A protein expression was detected in these samples and showed dark cytoplasm and nuclei with brown particles (Figure 7). The mean of total score including staining intensity and staining rate was 7.0556±2.5961 (Figure 8). We found low expression in 2 samples and high expression in 6 samples. Among the 8 samples, hMex-3A expression level (10.25) was the highest in the sample from non-invasive urothelial carcinoma. Mex-3A protein expression in para-cancerous tissue was also determined and it was 2.5126±1.1934. Mex-3A protein expression was significantly higher in cancerous tissue than in para-cancerous tissue (*P* <0.05).

## DISCUSSION

Mex-3 proteins play a role in core biological processes, such as embryonic development, epithelial homeostasis, immune responses, metabolism and cancer. For the time being, the Mex-3 family of proteins can adapt to function in complex and diverse fine-tuning regulatory mechanisms of different organisms by constituting a patterning gene. *hMex-3A* and 3B are also associated with Argonaute (Ago) proteins (Ago1 and Ago2) which are the key components of the RNA-induced silencing complex and have been implicated in tumor development [6]. Recently, it has been reported that knockdown of Mex3A by siRNAs inhibits cell proliferation and migration in human gastric cancer cells [7]. Like the regulatory role of CDX2 in gastrointestinal homeostasis and carcinogenesis, when human Mex3A was overexpressed in an intestinal cell line, impaired differentiation and altered polarity were observed with gain of stemness features [8]. Interestingly, maintenance of active germ cell mitosis by Mex-3 in C. elegans comprises repression of the cell-cycle inhibitor cyclin-dependent kinase inhibitor-2 (CKI-2) [9], which is orthologs of tumor suppressors. Recently, the functional role of Mex3C as a RNA-binding ubiquitin E3 ligase has been established, mediating the post-transcriptional decay of HLA-A allotypes [10]. It is necessary for normal postnatal growth to enhance the local expression of insulin-like growth factor 1 (IGF1) in bone [11] and IGF1 appears to be involved in metabolic regulation of energy balance [12]. By contrast, Mex3C was described as a new chromosomal instability (CIN) suppressor in CIN^+^ colorectal cancer [13]. Silencing of Mex3C leads to DNA replication stress, structural chromosome abnormalities, and chromosome mis-segregation. Moreover, Mex3 transcript levels are altered in several cancer types and are associated with disease, such as metabolic changes. Down regulation of murine Mex3C plays an important role in regulation of energy balance, because these mice exhibit reduced adipose deposition and increased energy expenditure [14]. Mex3C mutant mice also display a postnatal growth retardation phenotype; possibly due to a role for Mex3C in enhancing insulin-like growth factor 1 (IGF1) mRNA translation in developing bone, given that IGF1 is a primary mediator of growth hormone effects. Finally, polymorphic variations in human Mex3C may produce the susceptibility to essential hypertension [15]. hMex-3D mRNA also has been found in some cell lines and tissues. A variant form of Mex3D called TINO could negatively regulate the antiapoptotic protein BCL-2 in HeLa cells [16]. All the four proteins predominantly accumulate in the cytoplasm, and shuttle between the cytoplasm and the nucleus via the CRM1-dependent export pathway [17].

There are many treatment methods for bladder carcinoma due to its heterogeneity, so the conditions of metastasis, treatment response, relapse and clinical outcomes may vary remarkably among different individuals [18–20]. Therefore, it is necessary to understand biological properties and molecular mechanism of bladder carcinoma. According to the degree of tumor invasion into bladder wall, bladder carcinoma was divided into non-muscle invasive bladder carcinoma and muscle-invasive bladder carcinoma. Both the non-muscle-invasive bladder carcinoma cell line 5637 (the majority of the patients with bladder carcinoma have non-muscle-invasive cancer [21]) and the muscle-invasive bladder carcinoma cell line T24 were used in this study. As we know, cell line 5637 and T24 can be differentiated from normal bladder cell line. The former is characterized with its lower resistance to chemotherapy, and the latter could be distinguished by its abnormal mitochondrial metabolism. We measured *hMex-3A* mRNA expression in 5637 and T24 cells by real-time quantitative PCR. The results showed that *hMex-3A* mRNA expression was significantly higher in 5637 cells than in T24 cells. We also obtained the similar results in tissue samples of bladder carcinoma. Of the 8 patients with bladder carcinoma, one had non-invasive urothelial carcinoma (high-level) and 7 had invasive urothelial carcinoma. hMex-3A expression level was the highest in the sample from non-invasive urothelial carcinoma (10.25) among the 8 patients. We did not carry out statistical analysis about hMex-3A expression level in tissue samples because of too small number of cases. From these results, we can see that hMex-3A expression is similar in cell lines and tissue sample.

In recent years, RNA interference has been widely applied in exploring gene functions by down-regulating the expression of targeted genes. We used this technique to specifically silence *hMex-3A* expression in the bladder cancer cell lines 5637 and T24. Most notably, *hMex-3A* has been reported to be associated with Ago proteins ^7^. Ago proteins are components of the RNA-induced silencing complex and play a crucial role in RNA silencing [22, 23]. Recent studies have revealed that Ago1 and Ago2 play an important oncogenic role in breast and colon cancer [22, 23]. These repots suggest that *hMex-3A* may be associated with cancer development and progression. In this study, 5637 cell growth was significantly slower in *hMex*-3A-siRNA-transfected cells than in non-specific siRNA-transfected cells (*P* <0.05), indicating that 5637 cell line growth is associated with *hMex-3A*. More importantly, flow cytometry analysis displayed that apoptosis rate of 5637 cells was significantly higher in *hMex*-3A-siRNA-transfected cells than in non-specific siRNA-transfected cells (*P* <0.05), further confirming that 5637 cell line growth is associated with *hMex-3A*. In contrast, the effects of *hMex-3A* on T24 cells were different from that on 5637 cells. The inhibitory effect of *hMex-3A* on T24 cell growth was not marked. Also considering that *hMex-3A* expression level was significantly higher in 5637 cells than in T24 cells, we infer that different inhibitory effect of *hMex-3A* on 5637 and T24 cells may be associated with different expression levels in invasive and non-invasive bladder carcinoma. Our data imply that *hMex-3A* is strongly associated with 5637 bladder cancer cells.

In summary, silencing of *hMex-3A* resulted in a more obvious decrease in proliferation ability of 5637 than T24 bladder cancer cells, suggesting that a reduction in the *hMex-3A* expression level may lead to a more marked suppression of 5637 than T24 bladder cancer cell growth. This study provides a potential target for the treatment of bladder cancer.

The effects of hMex-3 proteins on human bladder tumorigenesis remain to be confirmed by clinical data. And tumorigenesis is a complicated process, so the exact mechanism by which *hMex-3A* contributes to tumorigenesis should be explored in the future.

## MATERIALS AND METHODS

All study methods were approved by the Ethics Committee of Shengjing Hospital of China Medical University.

### Analysis of TCGA database and Mex-3A gene screening

The data related to tumor are rich in TCGA database. We selected RNAseq and RNAseqV2 paired sample data for the analysis of expression level. For bladder carcinoma, there are a total of 19 paired-samples in TCGA database. Our analysis was based on the data of RNAseq of the 19 paired-samples.

### Specimens and cell culture

Human bladder cancer 5637 and T24 cells were obtained from the Shanghai Institute of Cell Biology, Chinese Academy of Sciences. T24 cell line with doubling time of 19h containing H-ras oncogene and tumor specific antigen expression was from a 68-year-old white woman with transitional cell carcinoma of bladder. While 5637 cell line was from a 68-year-old man with bladder carcinoma and it could produce SCF, IL-1, IL-3, IL-6, G-CSF, GM-CSF and so on. Cells were grown in Dulbecco’s modified Eagle medium (Gibco BRL/Life Technologies, Gaithersburg, MD, USA) with 100 IU/ml penicillin, 100 mg/ml streptomycin, and 10% heat inactivated fetal calf serum (PAA, Pasching, Austria) in a humidified atmosphere of 5% CO_2_ at 37°C. Typically, cells were seeded and maintained in the culture incubator for 12 h before the addition of other reagents.

### RT-PCR to select the cell line with highest level of Mex-3A mRNA from 5637 and T24 cell lines

Total RNA was extracted from the cultured cell lines using Trizol reagent (Invitrogen corp, Carlsbad, CA) and reverse-transcribed into cDNA using the M-MLV reverse transcriptase kit (Fermentas Inc, Hanover, MD). The following oligonucleotide primers were used to amplify the *Mex-3A* and *GAPDH* genes: *Mex-3A* forward, 5'-CGGAGTGGACTCTGGCTTTGAG-3' and reverse, 5'-CAGAGGAGAAGAGCACGGAGGT-3'; *GAPDH* forward, 5'-TGACTTCAACAGCGACACCCA-3' and reverse, 5'-CACCCTGTTGCTGTAGCCAAA-3'. Quantitative real-time PCR was performed with a Thermal Cycler Dice Real Time System (Takara, USA) and SYBR-Green I reagent (Takara, USA). Each sample was amplified in triplicate and the specificity was confirmed by dissociation analysis. The mRNA level of each sample was normalized to that of *GAPDH* prior to comparative analysis using the 2-^ΔCt^ method.

### Design and synthesis of sequences of siRNAs targeting hMex-3A

In order to suppress the *hMex-3A* expression in bladder cancer cells, *hMex-3A*- specific small interference RNAs (siRNAs) were chemically synthesized (GeneRay). The siRNA sequences were as follows: si-psc (sense, 5'- AGGCAAGGCTGCAAGATTAAGCTCGACTTAATCTTGCAGCCTTGCCT-3' and antisense, 5'-AGGCAAGGCTGCAAGATTAAGCTCGAGCTTAATCTTGCAGCCTTGCCT -3'). Irrelevant nucleotides not targeting any annotated human genes were used as the negative control as follows: si-NC (sense, 5'-CCGGTTCTCCGAACGTGTCACGTTTCAAGAGAA-3' and antisense, 5'- CGTGACACGTTCGGAGAATTTTTG-3'). After PCR identification and sequence analysis of positive clones, plasmids were extracted.

### Groups and cell transfection

Typically, 5637 and T24 cells were divided into 2 groups including negative control (non-specific siRNA-transfected cells) group and *hMex*-3A-siRNA-transfected cells group, respectively. Bladder cancer cells were seeded into 6 well plates for 24 h at a density of 2×105 cells per well. Cells covering 70– 80% of the hole wall were incubated in 2 ml of serum-free DMEM for 2 h followed by transfection according to the instructions of kit. Five hours later, cells were incubated in DMEM containing 10% fetal calf serum for 72 h followed by extraction of RNA.

### RT-PCR to observe Mex-3A mRNA levels in the post-transfected cells

Real time RT-PCR was performed with an ABI Prism 7700 Sequence Detection System (Applied Biosystems, Darmstadt, Germany) using the Quantitect SYBR Green PCR Kit from Qiagen (Hilden, Germany) following the manufacturer’s guidelines. Oligonucleotide primers were designed according to the sequences mentioned before. *hMex-3A* mRNA expression level was normalized to *GAPDH* expression in the cDNA preparation. The testing was performed in triplicate in each sample.

### Cell count analysis to assess the proliferation of bladder cancer cells

Following transfection with siRNAs at 1, 2, 3, 4, and 5 days, 5637 and T24 cells were harvested. Cell viability was measured using the Celigo Cell Counting (Nexcelom) according to the manufacturer's instructions. The testing was performed in triplicate in each sample.

### Cell apoptosis performed by flow cytometer

5637 cells were harvested 5 days after transfection, and then fixed with 1% paraformaldehyde for 20 min at room temperature followed by permeabilization with 0.1% Triton X-100 (Sigma) for 5 min on ice. Staining was performed with anti-Mex-3A antibody and visualized with goat anti-rabbit FITC-conjugated secondary antibody. For DNA content assessment, cells were incubated with a propidium iodide (50 mg/ml) and RNAse A (200 mg/ml) solution (Sigma) for 30 min at room temperature. Cell apoptosis was measured using the Annexin V-APC (eBioscience) according to the manufacturer's instructions. The testing was performed in triplicate in each sample.

### Immunohistochemical staining of hMex-3A in human bladder cancers

Samples of cancerous tissue and para-cancerous tissue (2 cm from cancerous tissue) were collected from 8 patients who underwent surgical treatment and diagnosed with bladder carcinoma by histopathology in our hospital between February 7, 2013 and March 18, 2016. The inclusion criteria were (1) primary bladder carcinoma; (2) no a history of chemotherapy and (3) definite diagnosis confirmed by pathology. According to pTNM stages, 2 patients were in Ta-T1, 3 patients in T2, 2 patients in T3 and one in T4.

Pathological sections were dried, treated using toluene, dehydrated using graded ethanol, treated using citrate buffer and sealed using goat serum. Rabbit hMex-3A (abcam) (1:200) was added in pathological sections and PBS was added in negative control sections followed by overnight at 4 °C. HRP-goat anti-rabbit secondary antibody was added in the sections for 30 min at 37 °C Followed by DAB coloration and nuclear staining.

Evaluation of staining intensity (0/1+/2+/3+) and staining rate: staining intensity including zero score (no staining), one score (weak staining), 2 scores (moderate staining) and 3 scores (strong staining); and staining rate including zero score (negative), one score (1-25%), 2 scores (26-50%), 3 scores (1-25%) and 4 scores (76-100%). Total score= score of staining intensity ×score of staining rate. The total score ≤ 6 scores were regarded as low expression and the total score > 6 score was regarded as high expression.

### Statistical analysis

Statistical analysis was performed using SPSS16.0 software. Each experiment was carried out at least three times, and all figures were showed by mean ± standard deviation ($\bar{x}\pm s$). Statistical analysis was performed using Student’s *t*-test. The criterion for statistical significance was set at *P* < 0.05.

## Abbreviations

siRNA: small interfering RNA
RBPs: RNA-binding proteins
siRNAs: small interference RNAs
CKI-2: cyclin-dependent kinase inhibitor-2
IGF1: insulin-like growth factor 1
CIN: chromosomal instability
IGF1: insulin-like growth factor 1
